# Decoding the Synaptic Proteome with Long-Term Exposure to Midazolam during Early Development

**DOI:** 10.3390/ijms23084137

**Published:** 2022-04-08

**Authors:** Nghi M. Nguyen, Neetha N. Vellichirammal, Chittibabu Guda, Gurudutt Pendyala

**Affiliations:** 1 Department of Anesthesiology, University of Nebraska Medical Center, Omaha, NE 68198, USA; minhnghi.nguyen@unmc.edu; 2 Department of Genetics, Cell Biology and Anatomy, University of Nebraska Medical Center, Omaha, NE 68198, USA; neethav@gmail.com (N.N.V.); babu.guda@unmc.edu (C.G.); 3 Child Health Research Institute, Omaha, NE 68198, USA

**Keywords:** alpha adducin, midazolam, NICU, neurodevelopment proteomics, synaptosomes

## Abstract

The intensive use of anesthetic and sedative agents in the neonatal intensive care unit (NICU) has raised controversial concerns about the potential neurodevelopmental risks. This study focused on midazolam (MDZ), a common benzodiazepine regularly used as a sedative on neonates in the NICU. Mounting evidence suggests a single exposure to MDZ during the neonatal period leads to learning disturbances. However, a knowledge gap that remains is how long-term exposure to MDZ during very early stages of life impacts synaptic alterations. Using a preclinical rodent model system, we mimicked a dose-escalation regimen on postnatal day 3 (P3) pups until day 21. Next, purified synaptosomes from P21 control and MDZ animals were subjected to quantitative mass-spectrometry-based proteomics, to identify potential proteomic signatures. Further analysis by ClueGO identified enrichment of proteins associated with actin-binding and protein depolymerization process. One potential hit identified was alpha adducin (ADD1), belonging to the family of cytoskeleton proteins, which was upregulated in the MDZ group and whose expression was further validated by Western blot. In summary, this study sheds new information on the long-term exposure of MDZ during the early stages of development impacts synaptic function, which could subsequently perturb neurobehavioral outcomes at later stages of life.

## 1. Introduction

Approximately 15 million babies are born prematurely each year globally [1,2], with most of them requiring surgery and mechanical ventilation to increase their survival rates. In the neonatal intensive care unit (NICU) setting, these preterm neonates are often treated for prolonged periods with sedative medications such as opioids, benzodiazepines, or ketamine to mitigate pain and reduce agitation [3,4]. The intensive use of analgesia and sedation in the NICU has raised concerns to the FDA about the potential implications for brain and cognitive development. Midazolam (MDZ) is a common benzodiazepine used in the NICU to relieve anxiety before undergoing major surgical procedures and as a drug to control seizure attacks. Previous studies have described MDZ sedation as contributing to spatial learning and memory impairments in vivo and disrupting synaptogenesis in vitro [5]. Importantly, it has been shown that a single exposure to MDZ with other anesthetic agents causes synaptic alterations and later causes learning disturbances in both clinical and preclinical models [4,5,6]. However, to date, there are no studies that have characterized how long-term exposure to MDZ during very early stages of development induces changes at the synaptic level. Accordingly, we employed high throughput quantitative mass-spectrometry-based proteomics on purified synaptosomes to identify synaptic protein signatures and functional pathways impacted by long-term MDZ exposure using a preclinical rodent model.

Synapses are junctions between neurons and glial cells that play a crucial role in the communication and transmission of information in the brain. Normal brain function and neural network development require precision control of the development and connectivity of the synapse [7]. Synaptosomes are organelles isolated from neuronal cells’ synaptic terminals [8,9]. The composition of synaptosomes includes the components of the presynaptic terminal, which consists of one or multiple mitochondria and synaptic vesicles, along with the postsynaptic membrane and postsynaptic density (PSD) [10]. A number of previous reports have used synaptosomes to investigate alterations in synaptic protein levels, activity, and localization within the neuronal compartment [8,11,12,13,14]. Since synaptosomes carry the morphological features and most of the chemical properties of the original nerve terminal, they have been a valuable tool for studying neurological diseases such as Alzheimer’s (AD), Parkinson’s (PD), and Schizophrenia [15]. In addition, synaptosomes isolated from the brain at certain regions are also applicable models for studying the relationship between structure and function in synaptic vesicle release [16].

Using a preclinical rodent model, we simulated long-term exposure to MDZ in a NICU setting by exposing the rat pups to MDZ in an escalating dose, from postnatal day (P)3 to P21. Then, we extracted the synaptosomes from P21 rat pups and used mass spectrometry to examine changes in the synaptic proteome. Further bioinformatics analyses, including ClueGO and IPA, were carried out to identify the biological process, molecular functions, and pathways associated with the identified differentially expressed proteins from mass spectrometry analysis. The proteomics-based approach used in this study allows for in-depth research into the changes in the synaptic signatures related to neonatal MDZ exposure. Further, identifying associated functional pathways and disease states affected by the protein expression changes in these synaptosomes reveals potential downstream effects that may continue to affect the development of MDZ-exposed neonates.

## 2. Results

Long-Term MDZ Exposure Alters the Synaptic Proteome

**Figure 1:**
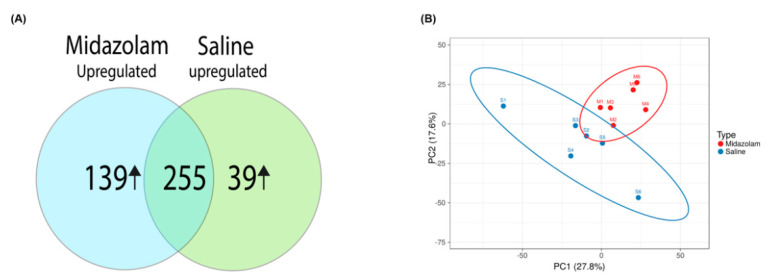
Overall data differentiation between control and MDZ-exposure samples: (**A**) Venn diagram showing total differentially expressed proteins found in between samples; the ↑ symbol represents the upregulation of DEPs with respect to saline or MDZ group. (**B**) principal component analysis (PCA) between the saline and midazolam-exposed samples from all six biological replicates from each group.

To ascertain if long-term MDZ exposure during early stages of life induced alterations in the synaptic proteome, we subjected P21 purified synaptosomes from the control and MDZ groups to high throughput quantitative mass-spectrometry-based proteomics. A total of 2262 proteins were identified. Further employing a criterion of 2+ unique peptides, and *p* < 0.05, we identified 433 proteins to be differentially expressed between the two groups (Table S1). Figure 1A shows the Venn diagram of the differentially expressed proteins after MDZ exposure based on a criterion of 1.5-fold up or down and *p* < 0.05. A total of 139 proteins were upregulated, while 39 were downregulated. Furthermore, principal component analysis (PCA) revealed good reproducibility of the biological replicates and overall separation between the groups (Figure 1B).

**Figure 2:**
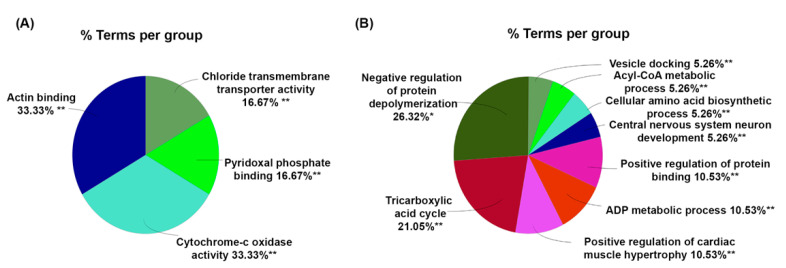
Mapping of molecular functions (**A**) and biological processes (**B**) using ClueGO. The asterisks represent the group term p-value representing each category. * *p* < 0.05 and ** *p* < 0.01.

Next, using the bioinformatics tool ClueGO, we analyzed the molecular functions and biological processes enriched with these DEPs (Figure 2). The most abundant biological process was negative regulation of protein depolymerization process with 26.32% of gene ontology (GO) terms associated with this process, followed by tricarboxylic acid cycle, ADP metabolic process, protein binding, etc. Interestingly, two enriched functional groups shared an equal 33.33% of the GO terms associated with actin-binding and cytochrome-c oxidative activity. Additionally, 16.67% of the GO terms related to DEPs were involved in pyridoxal phosphate binding. The remainder 16.67% were engaged in chloride transmembrane transporter activity. A list of GO terms and associated genes can be found in Table S2.

**Figure 3:**
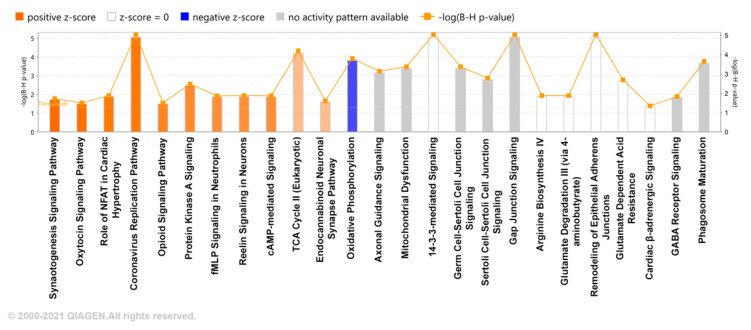
Ingenuity pathway analysis (IPA). Enriched pathways associated with long-term MDZ exposure. The pathways are ranked by the negative log of the FDR corrected *p*-value of the enrichment score and color-coded according to the Z score. A significantly increased pathway activity is indicated by a positive Z score, represented by the orange bars, and an overall decrease in pathway activity is represented by a negative Z score, represented by blue bars. The Gray bar represents enriched pathways with no predicted activity change.

We further investigated potential enriched pathways associated with the DEPs using ingenuity pathways analysis (IPA). As seen in Figure 3, pathways associated with synaptogenesis signaling, oxytocin signaling, and PKA signaling were enriched after MDZ exposure, while oxidative phosphorylation was downregulated. These data overall suggest that long-term exposure to MDZ treatment does have an impact on altering changes at the synapse by dysregulating key molecular and biological processes. Gene-to-disease associations are provided in Table S3.

**Figure 4:**
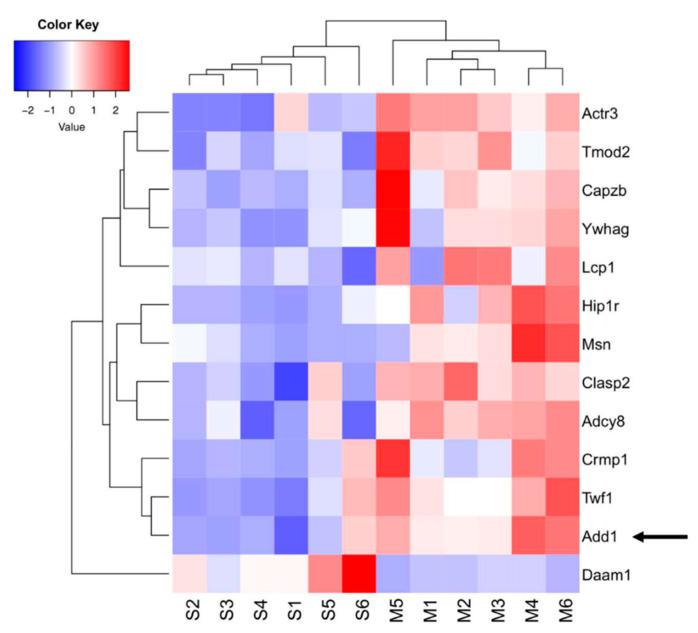
Heatmap visualization of differentially regulated proteins associated with actin-binding and negative regulation of protein depolymerization from ClueGO analysis features measured in the saline and midazolam-exposed samples from all six biological replicates from each group. The arrow highlights alpha-adducin (protein selected for post validation).

Since high throughput omics studies generally generate many potential hits, it is imperative to further validate them. Based on our ClueGO analysis that identified negative regulation of protein depolymerization process to be most abundant, we accordingly focused on validating hits associated with this function. We generated the heatmap of DEPs associated exclusively with the negative regulation of the protein depolymerization process and actin-binding function (Figure 4).

**Figure 5:**
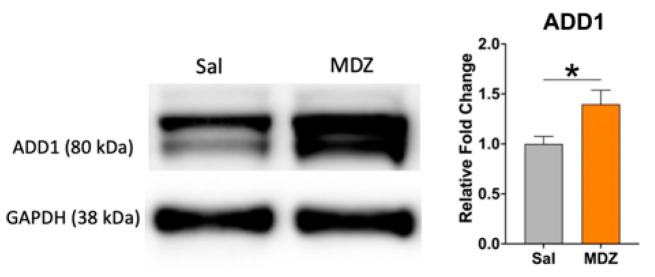
Validation of ADD1 upregulation after MDZ exposure. A representative Western blot is depicted here. GAPDH was used as an internal control. Data are represented as Mean ± SEM (n = 15/group) and significance was determined with an unpaired *t*-test after Welch’s correction. * *p* < 0.05.

One such hit we identified was alpha adducin (ADD1), which belongs to the cytoskeleton protein family. ADD1 was upregulated +1.75-fold in the MDZ group, and its expression level was further validated with Western blot (Figure 5). 

## 3. Discussion

In our current study, we show, for the first time, alterations in the synaptic proteome associated with long-term MDZ in a rodent model. Our main findings highlighted up- and downregulated differentially expressed proteins (DEPs) involved in various molecular functions (e.g., actin-binding, cytochrome c oxidase, pyridoxal phosphate binding) and biological processes (e.g., protein depolymerization, tricarboxylic cycle, central nervous system neuron development) with long-term exposure to MDZ. We also uncovered potential pathways associated with the DEPs such as synaptogenesis signaling, protein kinase A signaling, and oxidative phosphorylation. Altogether, these findings provide new insights pertaining to long-term exposure to MDZ during the early stages of development can impact neurodevelopmental outcomes, especially synaptic function.

The developing brain is vulnerable to constant exposure to neurotoxicity substances [17,18]. Previous studies have provided evidence showing anesthetics and sedative agents potentially modulate brain connectivity and neuron circuits [19,20,21]. The formation of neural circuits is driven by a process called synaptogenesis, which is highly dynamic and balances both synapse formation and elimination [22]. A study by De Roo et al. showed that mice that receive a single dose of MDZ at early development have a higher rate of synaptogenesis at postnatal days (P) 15, 20, and 30 [23]. One possible reason is that enhanced synaptogenesis could be a compensatory effect to aid in the possible loss of spines with a single acute dose of MDZ. Interestingly, a study by Xu et al. has revealed that neonate mice that received MDZ repetitively for five days have a lower synapse formed when these mice reach adulthood (P63) [5]. A potential explanation for this observation could be those multiple repetitive doses of MDZ could possibly induce more toxicity at the synapse, thus resulting in a lower count. These two studies point to the fact that exposure to long-term MDZ, albeit considering the number of exposures, can either increase synapse formation or lead to faster elimination during early development. Our findings here provide more support to the study by De Roo et al., based on our observation from our IPA analysis that shows enrichment of the synaptogenesis signaling pathway after MDZ exposure (Figure 3).

Learning and memory constitute significant aspects of neurodevelopment. Synaptic plasticity, which typically refers to the activity-dependent of strength and efficacy of synaptic transmission, is the feature that reflects learning and memory storage potential [24,25]. Earlier studies have suggested that exposure to MDZ during development subsequently leads to cognitive deficit and learning disturbances. Specifically, studies in rodents showed that exposure to anesthetic and sedative agents resulted in poor outcomes on cognitive tasks such as Morris water maze, radial arm maze, and Y-maze tests in the exposed animals [5,26]. Moreover, one recent clinical study showed that extremely preterm infants receiving opioids and benzodiazepines during their NICU stay were more likely to have lower cognitive, motor, and languages scores than infants with no exposure [27]. Altogether, these studies suggest potential alterations in synaptic plasticity with long-term exposure to sedatives and anesthetics.

A significant aspect associated with synapse function is the modulation of the actin cytoskeleton [28,29]. The actin cytoskeleton is essential to cellular processes involving membrane dynamics such as cell motility and morphogenesis [30]. Actin exists in monomeric globular (G-actin) and filamentous (F-actin) states [31]. The dynamic polymerization and depolymerization between G- and F-actin drive the morphological changes in dendritic spines that are associated with synaptic plasticity [32,33]. Actin regulators, including actin-binding protein (ABPs), can facilitate actin polymerization, promote disassembly, or stabilize filaments. Altogether, the dynamic actin cytoskeletons and regulation in dendritic spines development implies the notable area for focusing on the mechanism underlying abnormal or dysfunction of synapse formation upon the exposure to anesthetics and sedatives.

Our ClueGO analysis (Figure 2 and Table S2) identified molecular and biological processes associated with actin-binding and negative regulation of protein depolymerization to be significantly enriched after MDZ exposure. One critical potential hit we identified and further validated was alpha adducin or ADD1 (Figure 4 and Figure 5). In mammalian cells, the adducin family (alpha, beta, and gamma) is ubiquitously expressed. Multiple studies have highlighted the importance of adducin in neural cell signal transduction [34,35]. Adducin promotes the binding of actin to spectrin and may affect cytoskeleton transport, cell structure, and modulation of Na^+^/K^+^ pump activity [36,37]. In *Drosophila,* deletion of adducin results in an overgrowth of large-diameter presynaptic boutons, and an increase in synaptic retractions at the neuromuscular junction, while overexpression of adducin inhibits the formation of small-diameter type II and type III boutons [34,35]. Another study using the nematode *C. elegans* model implied that ADD1 contributes to learning and memorization. Deleting ADD1 in *C. elegans* subsequently impaired short- and long-term memory by destabilizing the actin at the synapse [38]. In the mice model, knockdown ADD1 interferes with the axon’s structure and integrity [39]. Our study observed an upregulation of ADD1 (Figure 5), which possibly implies stabilizing the cytoskeletal architecture perturbed by MDZ exposure. Future investigations into the functional role of ADD1 and the mechanisms associated with MDZ exposure are needed to establish the link between ADD1 and synaptic function.

Additionally, we found cytochrome c oxidase (COX)-related proteins, including Cox4i1, Cox5b, and Cox6c (Figure 2, Tables S1 and S2), are downregulated after MDZ exposure. Furthermore, those proteins are also associated with the deactivation of the oxidative phosphorylation pathways seen in IPA analysis (Figure 3, Table S3). Eukaryotic COX is the terminal enzyme associated with the energy-transducing mitochondrial electron transport chain [40]. COX locates the inner mitochondria membrane, facilitating the electrons transfer from reduced cytochrome c to molecular oxygen. COX also participates in proton pumping, which generates the electrochemical gradient for ATP synthesis [40,41]. Neurons’ activities and functions, including synapse formation, depend on ATP [42,43]. Notably, oxidative phosphorylation in the brain’s mitochondria generates and synthesizes approximately 90% of the ATP [44]. Synaptic mitochondria are critical for sustaining neurotransmission, and this process is controlled by energy metabolism, mitochondrial distribution, and trafficking, as well as cellular synaptic calcium flux [44,45,46]. Synaptic loss is an early but progressive pathological event in Alzheimer’s disease (AD) that causes cognitive impairment and memory loss, which is thought to be prevalent, especially in the later stages of the disease [46]. Interestingly, an association study explored the involvement of these COX-related genes in contributing genetic risk to developing AD in the Han Chinese population [47]. Taking our findings and the given relationship between COX and synaptic loss together, they can explain the impaired synaptic activity and possibly cognitive function seen with long-term exposure to anesthetics and sedatives.

In summary, our study elucidated a comprehensive characterization of the synaptic proteome, including yielding novel insights on how long-term exposure to MDZ during the early stages of development. Importantly, the identification of ADD1 as a potential target and further characterization of its downstream mechanisms can lend further insights into its role as a potential therapeutic to treat neurodevelopmental alterations associated with long-term MDZ use in neonates.

## 4. Materials and Methods

### 4.1. Animals

Pregnant dams—Pregnant Sprague Dawley rats were obtained from Charles River Laboratories Inc. (Wilmington, MA, USA) and grouped individually in a 12 h light–dark cycle. All animals were fed ad libitum and allowed to birth naturally. All procedures and protocols were approved by the Institutional Animal Care and Use Committee of the University of Nebraska Medical Center and conducted by the National Institutes of Health Guide for the Care and Use of Laboratory Animals.

### 4.2. Midazolam Treatment

**Figure 6:**
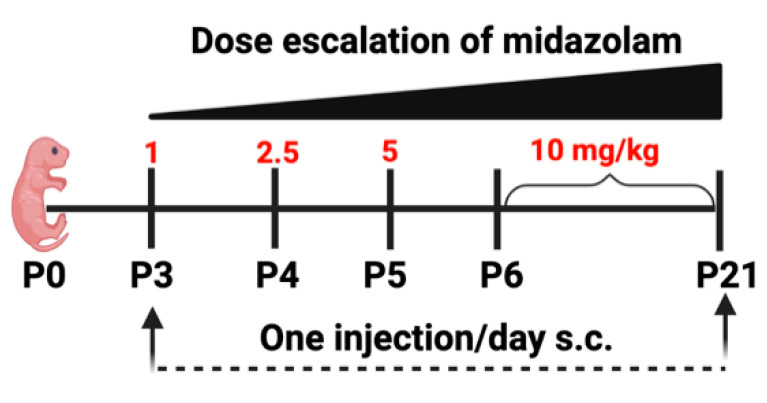
Schematic showing the dose-escalation regimen of midazolam treatment.

Starting at postnatal day 3 (P3), pups are given a single subcutaneous (s.c.) injection of 1 mg/kg midazolam (mixed with sterilized saline and administered at a uniform volume of 100 µL/10 g of birth weight of pup) and ramped up using a dose-escalation method until day 21 to closely mimic increments as performed in a NICU (Figure 6). Due to faster drug metabolism in rodents than in humans, a higher relative dose is required to induce an equivalent of 60 min sedation, which is similar to the NICU setting [6] This dosage was adapted based on previously published studies [5,6,26,48,49]. Immediately after injection, pups are monitored for any distresses, including placing them under a heating lamp to prevent thermal loss.

Pups were then evaluated for four reflex scales: posture, righting, cornea, and tail reflex, as described in [5], to determine the sedation status. The pups were monitored an additional 2–3 times throughout the day, to ensure that they were nursing post-treatment. For this study, pups were sacrificed at P21, and brains were harvested on ice and stored at −80 °C.

### 4.3. Purified Synaptosome Isolation

To investigate the effects of long-term midazolam exposure at early development on synaptic transmission, we isolated purified synaptosomes following the protocol previously described in our earlier publication [50]. Specifically, 100 mg of brain cortex was homogenized in 10 volumes of ice-cold homogenize buffer (0.32 M sucrose, 5 mM HEPES, 0.1 mM EDTA) containing protease–phosphatase inhibitors (Thermo Scientific, Waltham, MA, USA) with 12 strokes using Dounce Homogenizer Wheaton Overhead Stirrer (Wheaton, Millville, NJ, USA) at 250–300 rpm. The homogenized solution was spun at 1000× *g* for 10 min at 4 °C, and the supernatant was collected. A small aliquot of this homogenate was set aside, followed by centrifugation at 12,000× *g* for 20 min at 4 °C to obtain a crude synaptosome pellet. The crude synaptosome pellet was carefully resuspended in a homogenization buffer containing protease and phosphatase inhibitors, overlayed on top of sucrose gradients, and spun at 145,00× *g* for 1 h 40 at 4 °C using SW41 Ti Rotor (Beckman Coulter, Brea, CA, USA). The synaptosome band (approximately 1 mL) at the interface of 0.8 and 1.2 M sucrose was harvested using an 18-gauge needle and resuspended with a 9mL homogenization buffer. The solution was again spun at 14,500× *g* for 45 min at 4 °C using SW41 Ti Rotor (Beckman Coulter, Indianapolis, IN, USA), which resulted in the purified synaptosome pellet. This pellet was resuspended in 200 µL 1× PBS-containing protease–phosphatase inhibitors, followed by passing through a 27-gauge needle several times to be completely resuspended.

### 4.4. Mass Spectrometry Analysis

Protein quantification was performed using Pierce BCA protein assay (Thermo Scientific, Rockford, IL, USA), as described in our earlier studies [51,52,53,54,55]. The mass spectrometry analysis was performed by a UNMC Mass Spectrometry Core (Omaha, NE, USA), and the protocol was based on the label-free quantitative mass spectrometry protocol described in our recently published studies [55,56,57]. Specifically, 50 µg of protein per sample (n = 6/group) was subjected to chloroform–methanol extraction to remove the detergent in each sample. Prior to mass spectrometric analysis, the protein pellet was resuspended in 100 mM ammonium bicarbonate and digested with MS-graded trypsin (ThermoFisher, Waltham, MA, USA) overnight at 37 °C. The peptides were then cleaned using PepClean C18 spin columns (Thermo Scientific, Waltham, MA, USA) and resuspended in 2% acetonitrile (ACN) and 0.1% formic acid (FA). Then, 500 ng of each sample was loaded onto trap column Acclaim PepMap 100 75 µm × 2 cm C18 LC Columns (Thermo Scientific, Waltham, MA, USA), at a flow rate of 4 µL/min and then separated with a Thermo RSLC Ultimate 3000 (Thermo Scientific, Waltham, MA, USA) on a Thermo Easy-Spray PepMap RSLC C18 75 µm × 50cm C-18 2 µm column (Thermo Scientific, Waltham, MA, USA) with a step gradient of 4–25% solvent B (0.1% FA in 80 % ACN) from 10 to 130 min, and 25–45% solvent B for 130–145 min at 300 nL/min and 50 °C with a 180 min total run time.

The eluted peptides were then analyzed using a Thermo Orbitrap Fusion Lumos Tribrid (Thermo Scientific, Waltham, MA, USA) mass spectrometer in a data-dependent acquisition mode to analyze eluted peptides. A complete survey scan MS (from m/z 350 to 1800) was acquired in the Orbitrap with a resolution of 120,000. The AGC target for MS1 was set as 4 × 10^5^, and ion filling time was set as 100 ms. The most intense ions with charge state 2–6 were isolated in a 3 s cycle and fragmented using HCD fragmentation with 40% normalized collision energy and detected at a mass resolution of 30,000 at 200 m/z. The AGC target for MS/MS was set as 5 × 104 and ion filling time set at 60 ms; dynamic exclusion was set for 30 s with a 10 ppm mass window.

### 4.5. Protein Identification

We used the in-house mascot 2.6.2. (Matrix Science, Boston, MA, USA) search engine to further identify the proteins from MS/MS data, as described in our previous studies [55,56,57]. Specifically, MS/MS data were applied against the NCBI *Rattus norvegicus* protein. The search was set up for full tryptic peptides with a maximum of two missed cleavage sites. Acetylation of protein N-terminus and oxidized methionine were included as variable modifications, and carbamidomethylation of cysteine was set as a fixed modification. The precursor mass tolerance threshold was set at 10 ppm, and the maximum fragment mass error was 0.02 Da. The significance threshold of the ion score was calculated based on a false discovery rate (FDR) of ≤1%. Qualitative analysis was performed using progenesis QI proteomics 4.1 (Nonlinear Dynamics, Milford, MA, USA).

### 4.6. Bioinformatic Analysis

Proteins were identified as differentially expressed if the *t*-test *p*-value was ≤0.05, and absolute fold change was ≥1.5. In each comparison, heatmaps of all differentially expressed proteins were plotted using the function heatmap.2 in the R (version 3.6.0) package, *gplots*. Gene Ontology (GO) analysis of differentially expressed proteins was performed using the Cytoscape plug-in ClueGO [58]. Biological processes and molecular functions were included for GO enrichment analysis. Canonical pathway analysis was performed using the Ingenuity Pathway Analysis (IPA) software (Ingenuity^®^ Systems, Redwood City, CA, USA, www.ingenuity.com, accessed on 8 November 2021) by comparing the differentially expressed proteins against known canonical pathways (signaling and metabolic) within the IPA database. Enriched pathways with Benjamini–Hochberg false discovery rate (FDR) *p*-value ≤ 0.05 were considered for further analysis.

### 4.7. Western Blot

Western blotting was performed as described in our previous studies [51,56,59]. Briefly, purified synaptosomes (7.5 µg) from each animal from the two groups were loaded onto 10% Bis-Tris wells (Invitrogen, Waltham, MA, USA) under reducing conditions, followed by transfer to a nitrocellulose membrane using iBlot2 (Invitrogen, Waltham, MA, USA) and immunodetection. Nonspecific antibody blocking was performed using Superblock (ThermoFisher, Waltham, MA, USA). Immunoblotting was performed with primary antibodies overnight at 4 °C against ADD1 (1:1000, ProteinTech, Rosemont, IL, USA) and GAPDH (1:2500, Invitrogen, Waltham, MA, USA), followed by secondary antibody (1:2500, HRP-conjugated anti-rabbit IgG; Thermo Scientific, Waltham, MA, USA) and (1:2500, HRP-conjugated anti-mouse IgG; Thermo Scientific, Waltham, MA, USA) against ADD1 and GAPDH, respectively. Primary and secondary antibody dilutions were carried out according to the manufacturer’s suggestions. Blots were developed using Azure CSeries Imager (Azure Biosystems, Dublin, CA, USA) with SuperSignal West Pico Chemiluminescent Substrate (Thermo Scientific, Waltham, MA, USA).

### 4.8. Statistical Analyses

For proteomics analysis, after normalization, Student’s *t*-test was performed to identify proteins showing significant differences between groups (saline versus midazolam). Proteins that had at least two unique peptides and a *t*-test *p*-value < 0.05 were considered significant. All statistical tests were performed with GraphPad Prism version 8.4.3 (La Jolla, CA, USA). A *p*-value < 0.05 from an unpaired Student’s *t*-test, followed by Welch’s correction, was used to determine significance. Data are represented as the Mean ± SEM on the graphs.

## Data Availability

Data is contained within the article or supplementary material.

## Supplementary Materials

The following supporting information can be downloaded at: https://www.mdpi.com/article/10.3390/ijms23084137/s1.
