# Supplementary material for: Decoding the Synaptic Proteome with Long-Term Exposure to Midazolam during Early Development
Source: Int J Mol Sci. 2022 Apr 8;23(8):4137. doi: 10.3390/ijms23084137 (PMC9027542; doi:10.3390/ijms23084137)
Supplement: Supplementary file 1 [file ijms-23-04137-s001.zip › ijms-1662204-SF1.pdf]

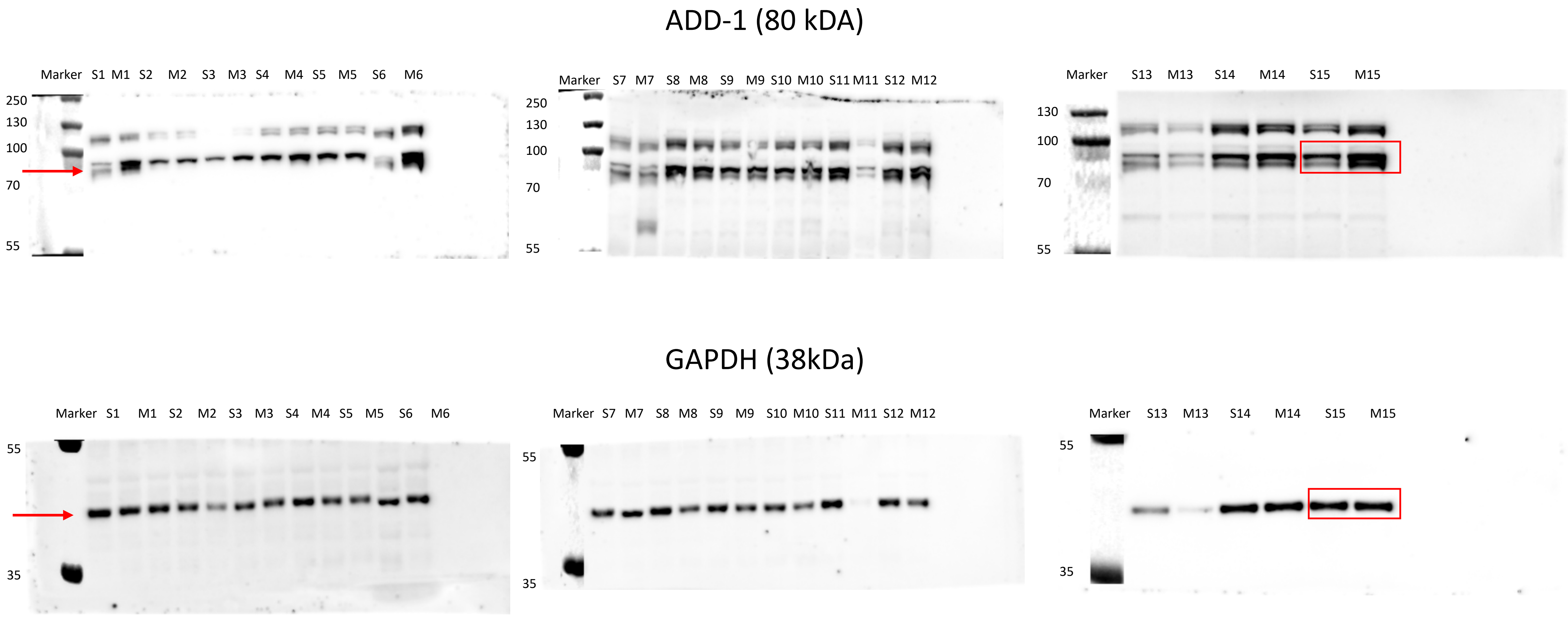

Supplementary Figure S1. Individual western blots on isolated purified synaptosomes from all animals used in the study. Boxed blots are shown in the manuscript. S-Saline, M-Midazolam
